# Circular RNA circRHOBTB3 is downregulated in hepatocellular carcinoma and suppresses cell proliferation by inhibiting miR-18a maturation

**DOI:** 10.1186/s13027-021-00384-1

**Published:** 2021-06-29

**Authors:** Gang Hu, Shusen Zhai, Sheng Yu, Zhen Huang, Ran Gao

**Affiliations:** 1 Department of General Surgery, Strategic Support Force Characteristic Medical Center, Beijing, 100101 People’s Republic of China; 2 Department of Oncology, Strategic Support Force Characteristic Medical Center, Beijing, 100101 People’s Republic of China; 3 Division of Hepatobiliopancreatic Surgery, Department of General Surgery, Nanfang Hospital, Southern Medical University, No. 1838 Guangzhou Avenue North, Guangzhou City, Guangdong Province 510515 People’s Republic of China

**Keywords:** Hepatocellular carcinoma, circRHOBTB3, miR-18a, Precursor

## Abstract

**Background:**

Circular RNA circRHOBTB3 has been characterized as a tumor suppressor in gastric cancer, while its role in hepatocellular carcinoma (HCC) is unknown. This study was carried out to analyze the role of circRHOBTB3 in HCC.

**Methods:**

In this study, circRHOBTB3, mature miR-18a, and miR-18a precursor in HCC and paired non-cancer tissues were detected by RT-qPCR. The role of circRHOBTB3 in the production of mature miR-18a was explored by transfecting circRHOBTB3 expression vector into HCC cells, followed by RT-qPCR to determine the expression of mature miR-18a and miR-18a precursor. The role of circRHOBTB3 and miR-18a in HCC cell proliferation was studied using CCK-8 assay.

**Results:**

CircRHOBTB3 was under-expressed in HCC compared to normal tissues. In HCC cells, circRHOBTB3 overexpression decreased mature miR-18a level but not miR-18a precursor. Cell proliferation analysis showed that circRHOBTB3 overexpression decreased cell proliferation while miR-18a overexpression increased cell proliferation. Moreover, circRHOBTB3 suppressed the role of miR-18a in cell proliferation.

**Conclusions:**

CircRHOBTB3 is downregulated in HCC and may suppress cell proliferation by reducing miR-18a production.

**Supplementary Information:**

The online version contains supplementary material available at 10.1186/s13027-021-00384-1.

## Introduction

Hepatocellular carcinoma (HCC) is a major malignancy that originated from the liver and accounted for more than 85% of all liver cancer cases [1]. The incidence of HCC varies greatly worldwide, with the highest incidence rate observed in African countries and eastern Asia [2, 3], mainly owing to the high prevalence of infections of hepatitis B virus (HBV) and hepatitis C virus (HCV) in these regions [4]. Especially in China, HCV and HBV affect about 30 million and 93 million people, accounting for about 9% of the Chinese population. As a result, about 53% HCC cases are believed to come from China [5, 6]. Therefore, preventative and treatment approaches for HCC are urgently needed.

Although HCV and HBV infections are the main risk factors for HCC, they are not sufficient for its occurrence and development [5, 6]. HCC is characterized by its hypervascularity and hepatocarcinogenesis. Receptor tyrosine kinases (RTKs) signaling pathways, such as Ras/Raf/MEK/ERK, Jak/Stat, and PI3K/AKT/mTOR, could activate many angiogenic signaling pathways, resulting in tumor cell proliferation, invasion, and metastasis [7, 8]. These data provide rational targets for innovative HCC therapies. mTOR inhibitors appear to be the most promising agents in this category [9–11]. Although many efforts have been made in molecularly targeted therapy in HCC, the effective targets for HCC remain lacking. As covalently closed RNAs with no or limited protein-coding capacity, circular RNAs (circRNAs) are emerging novel targets for targeted cancer therapy, mainly owing to their roles in regulating gene expression [12, 13]. In a recent study, Deng et al. reported a novel circRNA, circRHOBTB3 originated from exon 6 and exon 7 of RHOBTB3 gene as a tumor suppressor in gastric cancer [14]. Our preliminary microarray analysis revealed that circRHOBTB3 expression is altered in HCC (Fig.S1), and circRHOBTB3 is inversely correlated with miR-18a (data not shown).

miR-18a is one of the most conserved and multifunctional miRNAs in the polycistronic miR-17-92 cluster and is frequently overexpressed in malignant tumors [15]. Studies have shown that miR-18a is overexpressed in non-small-cell lung cancer (NSCLC) [16], cervical cancer (CC) [17], and gastric cancer (GC) [18]. Savitsky D et al. have verified that the 3′-UTR of IRF2 is a target of miR-18a, which can promote NSCLC development via downregulating IRF2 [19]. Mezache L et al. confirmed that miR-18a indirectly upregulates PD-L1 expression by activating the PI3K/AKT, MEK/ ERK, and Wnt/β-catenin pathways, resulting in CC progression [20]. In addition, another study showed that miR-18a is an oncogene and plays a vital role in GC development by negatively regulating the protein inhibitor of activated STAT (PIAS)-3 and thereby modulating STAT3 target genes [18]. Liu L et al. showed that miR-18a is upregulated in human HCC tissues and cell lines and promotes HCC cell proliferation and migration by targeting KLF4 and its p21 [21]. Despite emerging evidence about the role of miRNAs in cancer progression, fewer studies have explored the functions of miR-18a than other miRNAs in the miR-17-92 cluster in cancer development [15]. In this study, we focused on analyzing the crosstalk between circRHOBTB3 and miR-18a in HCC.

## Materials and methods

### HCC patients

A total of 62 HCC patients (38 to 66 years; median age: 52 years), including 42 males and 20 females, at Nanfang Hospital, Southern Medical University from May 2018 to May 2020 were enrolled in the study. These patients included 28 cases at AJCC stage I or II and 34 cases at III or IV. HBV infection was observed in 22 cases, and HCV infection was observed in 30 cases. To exclude other factors that may affect the expression of target genes, patients with initiated therapy or other severe clinical disorders, such as diabetes, heart diseases, severe infections, and other malignancies were excluded. The study was approved by the Ethics Committee of Nanfang Hospital. All patients signed the informed consent.

### HCC tissue collections and HCC cell lines

HCC and adjacent non-cancer tissues were collected by aspirating 3 times in different directions using fine needles from all patients prior to therapies. Tissue samples were confirmed histopathologically using hematoxylin-eosin (H&E) staining and stored in liquid nitrogen prior to the subsequent assays.

SNU-449 and SNU-387 (ATCC) cell lines were used as HCC cell models in this study. Normal liver THLE-2 cell line (ATCC) was used as the control. Cells were cultured in RPMI-1640 medium with 10% FBS at 37 °C and 5% CO_2_.

### Lipofectamine 2000-mediated transfections

CircRHOBTB3 expression vector was established with pcDNA3.1 (Addgene) as the backbone. MiR-18a mimic and negative control (NC) were purchased from Sigma-Aldrich. To overexpress circRHOBTB3 and miR-18a, SNU-449 and SNU-387 were transfected with either 1 μg expression vector or 40 nM miRNA using Lipofectamine 2000 (Invitrogen). Empty vector or NC miRNA transfected cells were used as the NC cells.

### RNA preparations

RNAs were extracted from SNU-449 and SNU-387 cells and tissues collected from the 62 HCC patients using Ribozol reagent (VWR Life Science) and treated with DNase I (Invitrogen) for 1 h at 37 °C to completely remove genomic DNAs. RNA integrity was examined by electrophoresis on 6% urea-PAGE gel, and RNA purity was reflected by OD260/280 ratio.

### qRT-PCR

For mRNA detection, RNA samples were reverse transcribed (RTs) using the SSRT-III-RT system (Invitrogen) and subjected to qPCR analysis with LightCycler® 480 SYBR Green I Master (Roche Life Science). The relative mRNA expression was determined using the 2^-△△Ct^ method with GAPDH as the reference. The PCR primers were circRHOBTB3 forward 5′-GAAGTTGAAAGATTCTGGGGA-3′ and reverse 5′-ACTGGCAGCAGAACAGCAAG-3′ and GAPDH forward 5′-GTCTCCTCTGACT TCAACAGCG-3′ and reverse 5′-ACCACCCTGTTGCTGTAGCCAA-3′. The levels of miR-18a precursor and mature miR-18a were examined by qRT-PCRs using All-in-One™ miRNA qRT-PCR Detection Kit (GeneCopoeia). The relative miRNA expression was determined using the 2^-△△Ct^ method with U6 as the reference. The PCR primers were miR-18a precursor forward 5′-TGTTCTAAGGTGCATCTAG-3′ and reverse 5′-TGCCAGAAGGAGCACTTAG-3′, miR-18a forward 5′-CACGCATAAGG TGCATCTAGTGC-3′ and reverse 5′-CCAGTGCAGGGTCCGAGGTA-3′, and U6 forward 5′-CTCGCTTCGGCAGCACA-3′ and reverse 5′-AACGCTTCACGAATTTG CGT-3′. All qPCRs were performed in three technical replicates.

### Cell proliferation assay

At 48 h of post-transfection, cell proliferation was analyzed using CCK-8 kits (Sigma-Aldrich). In brief, 5000 cells were placed in each well of 96-well plates. Cell proliferation was measured every 24 h for 4 times at 2 h after the addition of 10% CCK-8 by measuring the optical density (OD) at 450 nm.

### Statistical analysis

CircRHOBTB3 and miR-18a expression in tissues and cell lines fulfilled the normal distribution, which was analyzed by the Kolmogorov-Smirnov test in SPSS software. Thus, circRHOBTB3 and miR-18a expression in tissues from HCC patients (*n* = 62) were compared by paired t test. Comparisons between two independent groups were performed by unpaired t test. ANOVA Tukey’s test was used to compare differences among multiple transfection groups. *P* < 0.05 was statistically significant.

## Results

### HCC exhibited downregulated circRHOBTB3 expression

**Fig. 1:**
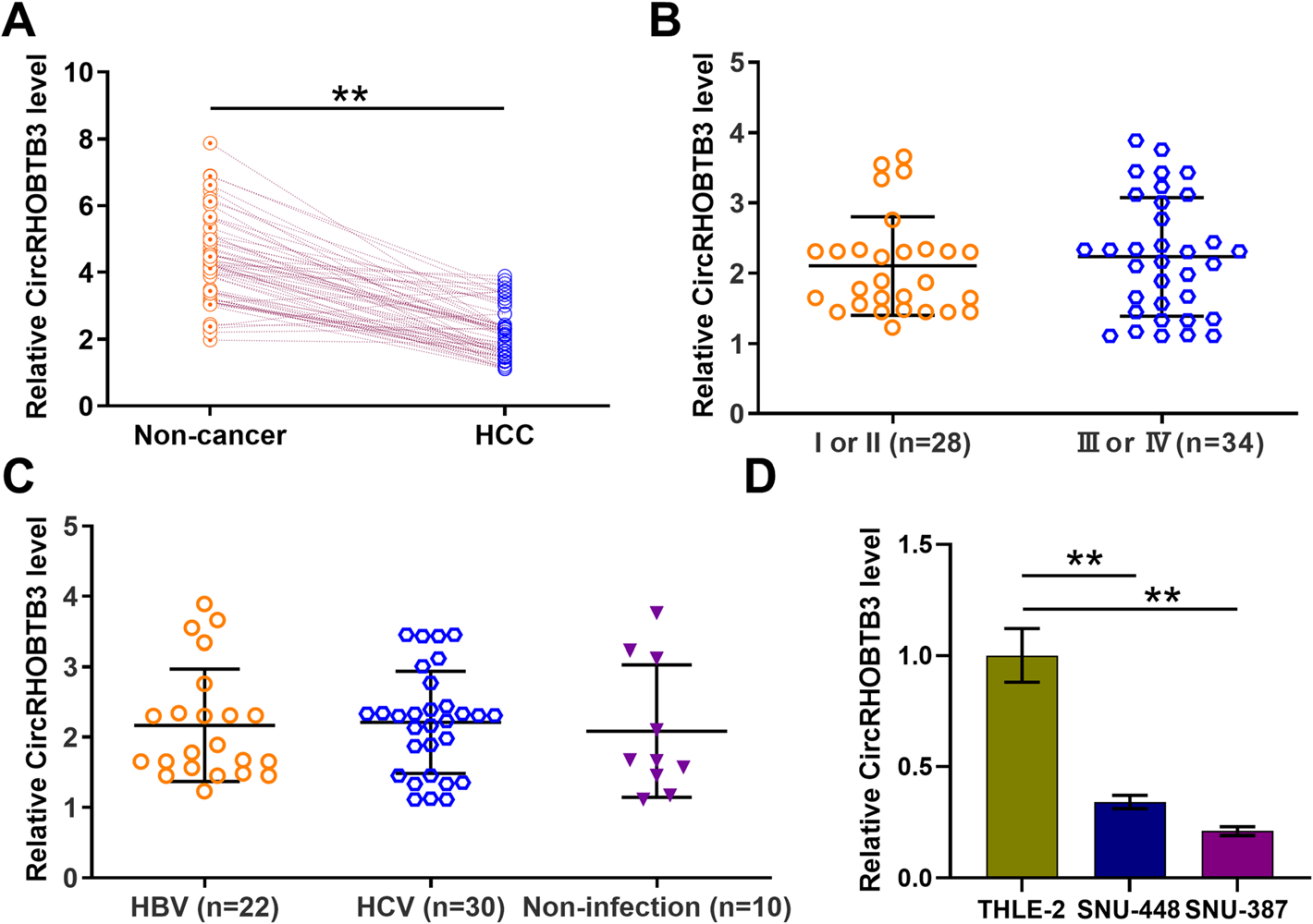
CircRHOBTB3 expression was downregulated in HCC but was not affected by clinical stages and infections of HBV and HCV. CircRHOBTB3 expression in paired HCC and non-tumor tissues from HCC patients (*n* = 62) was analyzed by qRT-PCR (**A**). CircRHOBTB3 expressions at AJCC stage I or II and stage III or IV HCC tissues were compared (**B**). CircRHOBTB3 expressions were compared in HCC tissues from patients with HCV infection, HBV infection, and non-infection (**C**). CircRHOBTB3 levels in SNU-448, SNU-387, and normal liver cell line THLE-2 were measured by qRT-PCR (**D**). CircRHOBTB3 levels were expressed as the average of three technical qRT-PCR replicates in tissue samples from HCC patients (*n* = 62) or in cell lines. GAPDH was used for normalization. ** *p* < 0.01

CircRHOBTB3 expression in paired tissues from HCC patients (*n* = 62) was analyzed by RT-qPCR. CircRHOBTB3 was significantly under-expressed in HCC tissues (Fig. 1A, *p* < 0.01). CircRHOBTB3 expression in HCC tissues was not significantly different between AJCC stages (I or II vs. III or IV; Fig. 1B, *p* < 0.01). Among the HCC patients, HBV infection was observed in 22 cases, HCV infection was observed in 30 cases, and non-infection was observed in 10 cases. No significant differences in circRHOBTB3 expression were found among these three groups (Fig. 1C). CircRHOBTB3 levels in SNU-448, SNU-387, and normal liver cell line THLE-2 were measured by RT-qPCR. The results showed that circRHOBTB3 was also downregulated in SNU-448 and SNU-387 cell lines compared to normal THLE-2 cell line (Fig. 1D). It is worth noting that RHOBTB3 mRNA was also downregulated in HCC cell lines (Fig. S2).

### Both mature miR-18a and miR-18a precursor were upregulated in HCC, but circRHOBTB3 was only inversely correlated with mature miR-18a

**Fig. 2:**
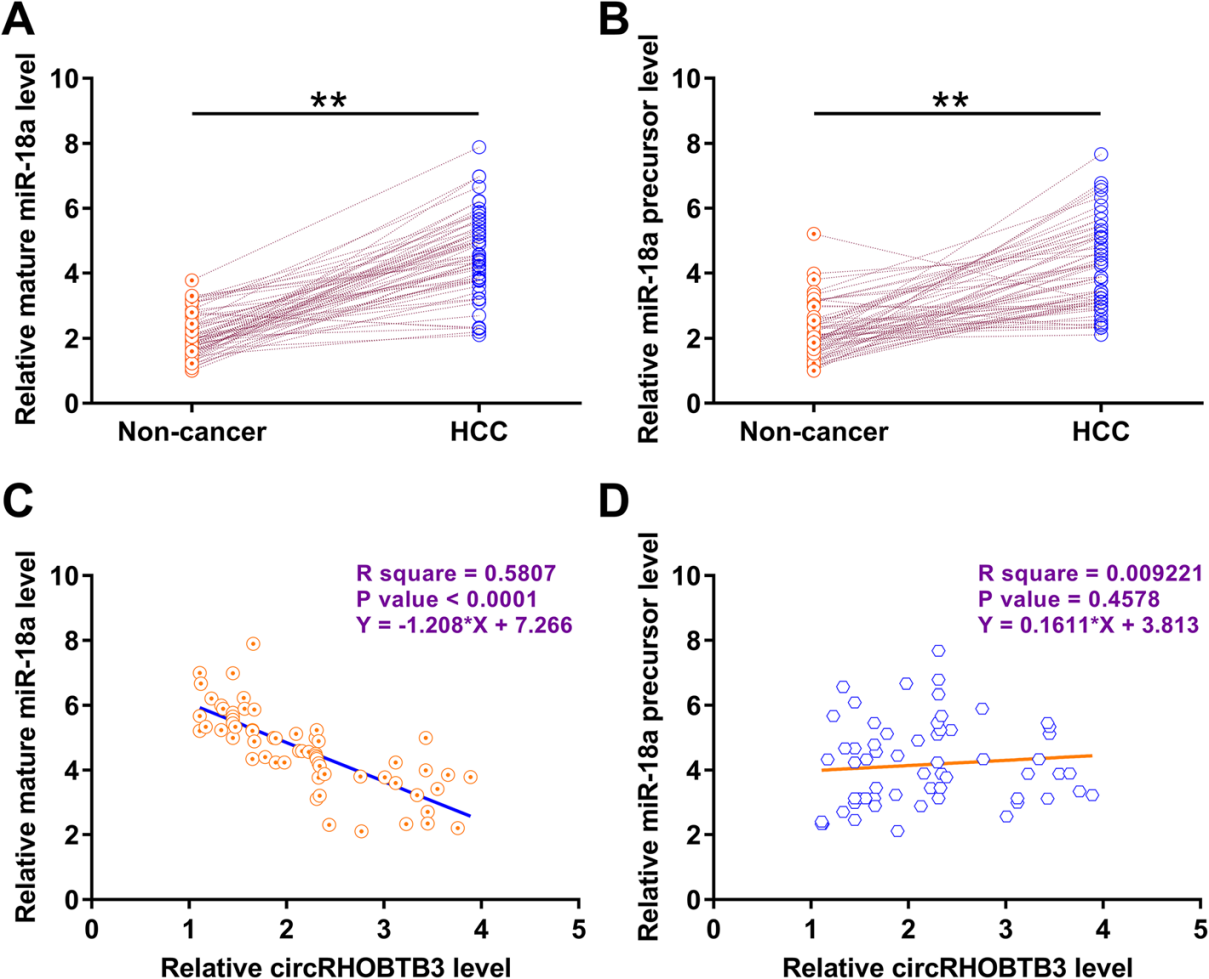
Mature miR-18a and miR-18a precursor were both upregulated in HCC, but circRHOBTB3 was only inversely correlated with mature miR-18a. Mature miR-18a (**A**) and miR-18a precursor (**B**) expressions in paired tissues from HCC patients (*n* = 62) were analyzed by qRT-PCRs. Expression levels of mature miR-18a and miR-18a precursor in tissue samples from HCC patients (*n* = 62) were expressed as the average of three technical qRT-PCR replicates. U6 was used for normalization. ** *p* < 0.01. Correlations between circRHOBTB3 and mature miR-18a (**C**) or miR-18a precursor (**D**) were analyzed by Pearson’s correlation coefficient

Mature miR-18a and miR-18a precursor expressions in paired tissues from HCC patients (*n* = 62) were also analyzed by RT-qPCRs. Mature miR-18a (Fig. 2A) and miR-18a precursor (Fig. 2B) were significantly overexpressed in HCC tissues than in non-tumor tissues (*p* < 0.01). Correlation analysis performed by Pearson’s correlation coefficient showed thatcircRHOBTB3 was inversely correlated with mature miR-18a (Fig. 2C), but not miR-18a precursor (Fig. 2D) across HCC tissues. Therefore, we hypothesized that circRHOBTB3 is likely involved in miR-18a maturation.

### CircRHOBTB3 overexpression decreased the production of mature miR-18a

**Fig. 3:**
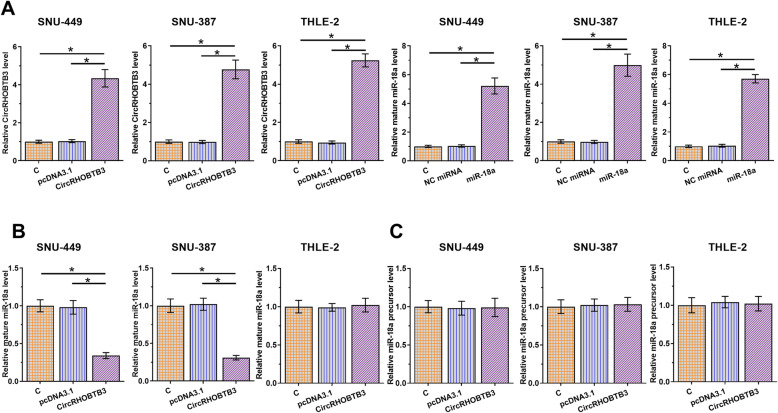
**CircRHOBTB3 overexpression decreased mature miR-18a level in SNU-449 and SNU-387 cells.** To study the role of circRHOBTB3 in regulating miR-18a maturation, SNU-449, SNU-387, and THLE-2 cells were transfected with circRHOBTB3 expression vector or miR-18a mimic, followed by qRT-PCRs to confirm the overexpression of circRHOBTB3 and mature miR-18a (**A**). The effects of circRHOBTB3 overexpression on mature miR-18a (**B**) and miR-18a precursor (**C**) were also analyzed by qRT-PCR. Expressions of circRHOBTB3, mature miR-18a, and miR-18a precursor were expressed as the average of three technical qRT-PCR replicates. GAPDH was used for circRHOBTB3 normalization, and U6 was used for mature miR-18a and miR-18a precursor normalization. * *p* < 0.05

To study the role of circRHOBTB3 in regulating miR-18a maturation, SNU-449 and SNU-387 cells were transfected with circRHOBTB3 expression vector or miR-18a mimic, followed by RT-qPCRs to confirm the overexpression of circRHOBTB3 and mature miR-18a (Fig. 3A, *p* < 0.05). CircRHOBTB3 overexpression significantly decreased the level of mature miR-18a (Fig. 3B, *p* < 0.05), but not miR-18a precursor (Fig. 3C). Therefore, circRHOBTB3 may suppress miR-18a maturation in HCC cells. In addition, circRHOBTB3 had no effect on the levels of mature miR-18a and miR-18a precursor in THLE-2 cell lines (Fig. S2).

### CircRHOBTB3 overexpression decreased HCC cell proliferation

**Fig. 4:**
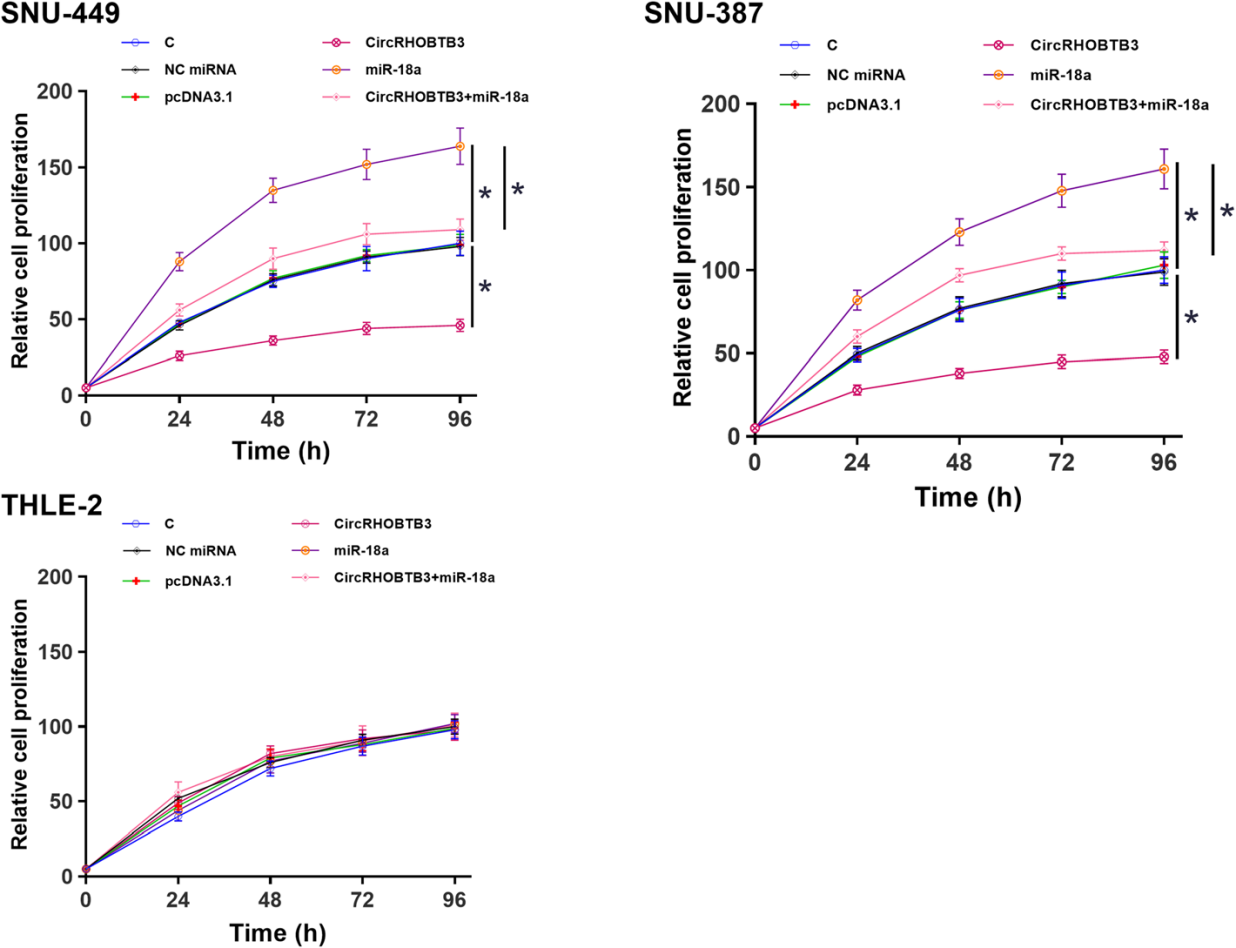
CircRHOBTB3 overexpression decreased SNU-449 and SNU-387 cell proliferation via miR-18a. CCK-8 assay was performed to study the role of circRHOBTB3 and miR-18a in regulating the proliferation of SNU-449, SNU-387, and THLE-2 cells. * *p* < 0.05

The role of circRHOBTB3 and miR-18a in the proliferation of SNU-449 and SNU-387 cells was analyzed by CCK-8 assay. CircRHOBTB3 overexpression decreased cell proliferation, while miR-18a overexpression increased cell proliferation. Moreover, circRHOBTB3 suppressed the role of miR-18a in cell proliferation (Fig. 4, *p* < 0.05). However, CircRHOBTB3 and miR-18a overexpression have no effect on the proliferation of THLE-2 cell lines (Fig. S2).

## Discussion

We analyzed the differential expression of circRHOBTB3 in HCC and studied its crosstalk with miR-18a. The results showed that circRHOBTB3 was under-expressed in HCC and inhibited HCC cell proliferation by suppressing miR-18a maturation.

CircRHOBTB3 is significantly downregulated in gastric cancer [14]. In addition, circRHOBTB3 overexpression sponges miR-654-3p to suppress the growth of gastric tumor, suggesting the role of circRHOBTB3 as a tumor suppressor in this malignancy [14]. This study, for the first time, reported circRHOBTB3 downregulation in HCC. Interestingly, circRHOBTB3 expression was not significantly affected by clinical stags, suggesting that circRHOBTB3 may participate in the whole process of HCC development but not specific stages. Moreover, circRHOBTB3 expression was also not significantly affected by HBV and HCV infections, the major risk factors for HCC [5, 6]. Therefore, circRHOBTB3 may participate in HCC through HBV- and HCV-independent pathways.

MiR-18a has been characterized as an oncogenic miRNA in many cancers, including HCC [21]. MiR-18a is overexpressed in HCC and targets KLF4 to promote tumor metastasis and growth in HCC [21]. Consistently, our study confirmed the upregulation of both miR-18a precursor and mature miR-18a in HCC. Therefore, the miR-18a may be upregulated at the transcription level from the process from pri-miRNA to miRNA precursor.

Besides regulating gene expression at transcriptional and translational levels, circRNAs may also sponge miRNAs to participate in human cancers, including HCC [22]. For instance, circRNA circMTO1 may sponge miR-9 to inhibit the progression of HCC [22]. In this study, we reported the interaction between circRHOBTB3 and miR-18a. Interestingly, circRHOBTB3 is unlikely a sponge of miR-18a. Memczak S et al. found that CDR1as can function as a miR-7 sponge to reduce midbrain sizes in zebrafish, and this biological activity of CDR1as could be partially rescued by injecting miR-7 precursor [23]. Our results showed that circRHOBTB3 overexpression decreased mature miR-18a level, leading to reduced HCC cell proliferation while had no effect on miR-18a precursor level. These results suggested that the effect of circRHOBTB3 overexpression on HCC cell proliferation cannot be rescued by the miR-18a precursor. However, our results showed that the effect of circRHOBTB3 on HCC cell proliferation could be rescued by mature miR-18a overexpression. Therefore, we believe that circRHOBTB3 overexpression-reduced HCC cell proliferation may be due to its role to suppress miR-18a maturation rather than its role as a molecular sponge of miR-18. Our findings revealed another type of interaction between circRHOBTB3 and miR-18a and suggested that circRHOBTB3 overexpression hinders the formation of mature miR-18a instead of miR-18a precursor, thereby reducing HCC cell proliferation. Because the formation of mature miRNAs requires transportation of miRNA precursors from the nucleus to the cytoplasm, we speculate that circRHOBTB3 may be involved in forming mature miRNAs from miRNA precursors. Our future studies will explore this possibility. Interestingly, we did not observe the increased level of miR-18a precursor along with decreased mature miR-18a in cells with circRHOBTB3 overexpression. It possibly indicated that circRHOBTB3 could also downregulate total miR-18a. In addition, we found that RHOBTB3 mRNA level was downregulated in HCC line cells. MiRNAs regulate physiological and pathological processes via inhibiting target mRNA translation or promoting mRNA degradation [24]. In this study, we only revealed the interaction between circRHOBTB3 and miR-18a. The underlying mechanism for this interaction needs to be further explored.

## Conclusion

In conclusion, circRHOBTB3 is under-expressed in HCC. Moreover, circRHOBTB3 may suppress the maturation of miR-18a to promote HCC cell proliferation.

## Supplementary Information

**Additional file 1 Fig. S1** The microarray results of circRHOBTB3 and miR-18a

**Additional file 2 Fig. S2** RHOBTB3 mRNA expression in cell lines.

## Data Availability

The data that support the findings of this study are available on request from the corresponding author: Sheng Yu, Division of Hepatobiliopancreatic Surgery, Department of General Surgery, Nanfang Hospital, Southern Medical University, No. 1838 Guangzhou Avenue North, Guangzhou City, Guangdong Province, 510,515, P. R. China. The data are not publicly available due to their containing information that could compromise the privacy of research participants.
