# Supplementary figures and images for: Circular RNA circRHOBTB3 is downregulated in hepatocellular carcinoma and suppresses cell proliferation by inhibiting miR-18a maturation
Source: Infect Agent Cancer. 2021 Jun 29;16:48. doi: 10.1186/s13027-021-00384-1 (PMC8243428; doi:10.1186/s13027-021-00384-1)

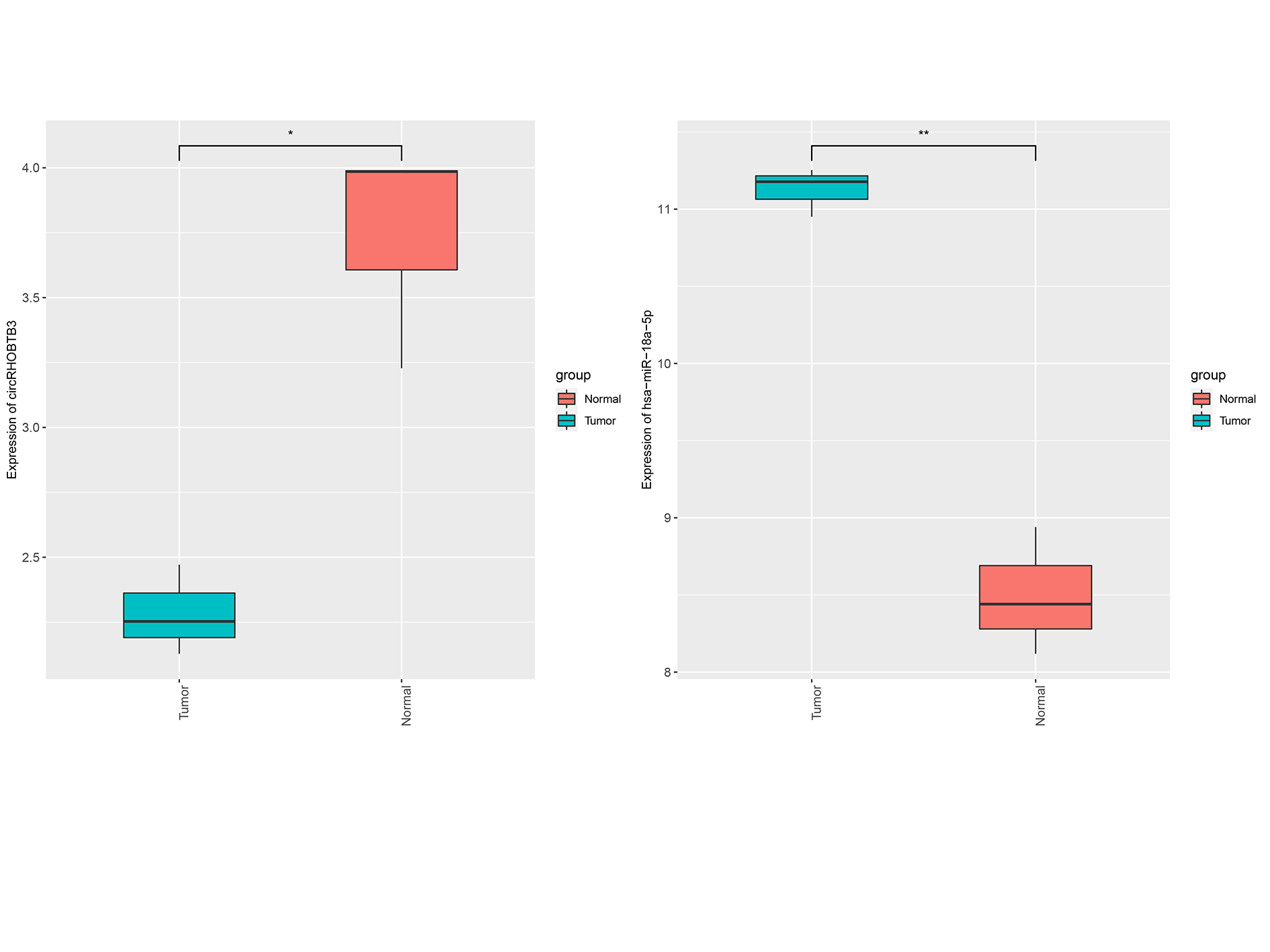

Supplement: Supplementary file 1 — Additional file 1 Fig. S1 The microarray results of circRHOBTB3 and miR-18a [file 13027_2021_384_MOESM1_ESM.tif]

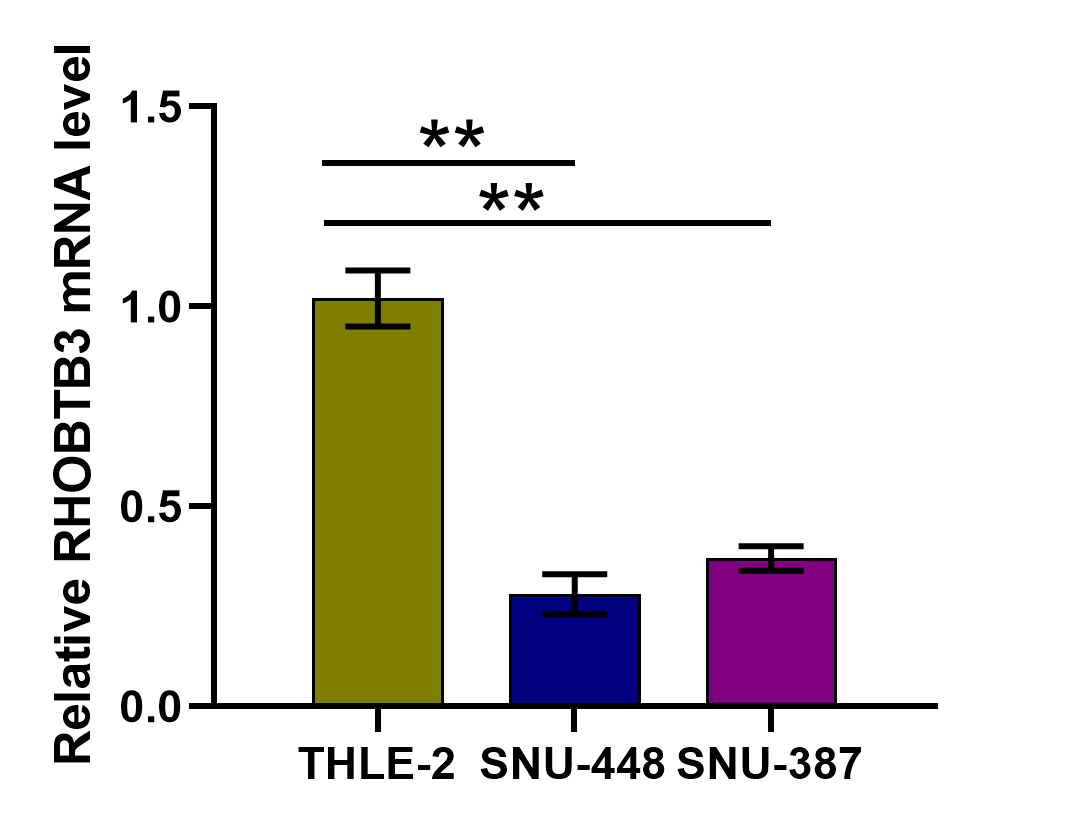

Supplement: Supplementary file 2 — Additional file 2 Fig. S2 RHOBTB3 mRNA expression in cell lines. [file 13027_2021_384_MOESM2_ESM.tif]
